# Thermocouple-integrated resonant microcantilever for on-chip thermogravimetric (TG) and differential thermal analysis (DTA) dual characterization applications

**DOI:** 10.1038/s41378-024-00828-9

**Published:** 2025-03-26

**Authors:** Yuhang Yang, Hao Jia, Zechun Li, Zhi Cao, Haozhi Zhang, Pengcheng Xu, Xinxin Li

**Affiliations:** 1 State Key Lab of Transducer Technology, Shanghai Institute of Microsystem and Information Technology, Chinese Academy of Sciences, 200050 Shanghai, China; 2 University of Chinese Academy of Sciences, 100049 Beijing, China; 3 School of Chemical and Environmental Engineering, Shanghai Institute of Technology, 201418 Shanghai, China

**Keywords:** Electrical and electronic engineering, Chemistry

## Abstract

This work presents an integrated microsensor that combines the dual characterization capabilities of thermogravimetric analysis (TGA) and differential thermal analysis (DTA). We integrated two pairs of thermocouples, heating resistors, and resonant drive/detection resistors into a single microcantilever, where the cantilever resonant frequency shifts respond to the mass change and the output voltage of the integrated thermocouples respond to the sample temperature. This integration enables programmable temperature control, temperature variation, and mass detection on a single chip. Our chip can achieve heating and cooling rates above 600 °C/min, which is significantly faster than commercial instruments with satisfactory measurement accuracy. The integrated polysilicon thermocouples bring high power responsivity of 6 V/W, making them suitable for highly sensitive DTA measurements on a chip. Moreover, the cantilever offers picogram (10^–12^g) level mass resolution, reducing sample consumption from milligrams to nanogram levels. Additionally, the on-chip sample heating allows for easy observation of sample morphological evolution during heating under an optical microscope. We validated the dual functionality by conducting TGA measurements on a standard sample of calcium oxalate monohydrate (CaC_2_O_4_ ∙ H_2_O) and DTA measurements on high-purity indium (In) and tin (Sn). The results indicate consistent measurements with the true values of the standard sample and high measurement efficiency. Our integrated cantilever chip is anticipated to have broad applications in high-performance and efficient TGA and DTA characterization.

## Introduction

Thermal analysis technologies evaluate temperature-dependent properties of substances under controlled conditions and are widely used in chemical engineering, food safety, and drug analysis^1–6^. Among the established thermal analysis techniques, thermogravimetric analysis (TGA) and differential thermal analysis (DTA) stand out as two widely used characterization methods^5–7^. TGA is a classic characterization technique for measuring the mass change of materials during programmed heating in a particular atmosphere and is frequently used to investigate the thermal stability of materials as well as the thermal properties of materials such as evaporation, decomposition, dehydration, and oxidation during the heating process^8,9^. DTA primarily measures the temperature shifts of material in programmed temperatures, enabling the investigation of the endothermic or exothermic properties of materials during phase transitions or chemical reactions^7,10^. Therefore, the combined characterization of TGA and DTA techniques is conducive to establishing comprehensive, multidimensional thermal properties of materials^11–13^, which is of great significance for the rapidly advanced new functional materials and related research fields^13,14^.

Although TGA and DTA instruments have been commercialized for decades, their inherent shortcomings have become increasingly apparent with the rapid advancement of new functional materials and related research fields^15–17^. Due to the bulk size of commercial DTA instruments, a large heat capacity is introduced, resulting in a power response typically in the mV/W level^18–20^. It is, therefore, essential to ensure that the sample consumption of commercial DTA instruments is not too small in order to guarantee sufficient DTA signal amplitude. Typically, this should be on the milligram level, as evidenced by previous studies^21–24^. Meanwhile, the thermal balances used in current commercial instruments normally have mass sensitivities on the sub-microgram scale^7,25–27^, so mg-level samples are also required for commercial TGA measurement. The available commercial DTA and TGA instruments usually employ a crucible to hold the sample, preventing direct contact between the temperature measuring element and the sample itself, which introduces an inaccuracy in the temperature measurement. However, at high heating rates, mg-scale samples are difficult to heat uniformly, and a wide temperature distribution occurs, causing the TGA and DTA signals of the samples to have a hysteresis relative to the true values, and this hysteresis becomes more significant as the heating rate increases^28–30^. Hence, commercial instruments typically limit the heating rate to less than 100–200 °C/min to ensure measurement accuracy^17,18^, resulting in time-consuming traditional TGA/DTA measurement, negatively affecting the efficiency of new materials development. Furthermore, the high sample volume of current commercial instruments is not conducive to measuring expensive, small batches of materials such as new drugs. Using a closed bulk furnace by commercial instruments to heat the sample presents a challenge in integrating the microscope for in situ optical observation of sample appearance^15,31^. We note that based on MEMS (micro-electromechanical system) technology, many pairs of thermocouples have been integrated into chips, enabling on-chip DTA analysis capabilities^15,21,22,24^. However, such chips are unable to accurately measure the mass of the sample, let alone perform on-chip TGA analysis. Our group initially used a micro-resonant cantilever integrated with on-chip heating and mass measurement functions, which enabled the on-chip TGA measurement of samples at the nanogram level^32–35^. However, the cantilever can only determine the overall temperature by measuring the change in the resistance of the heater and cannot accurately measure the sample temperature. To date, the available MEMS sensors can only measure TGA or have DTA capabilities, but they have not yet been able to perform both functions on a single chip.

Herein, an integrated resonant microcantilever is proposed and developed to achieve dual thermal functions of TGA and DTA on a single MEMS chip. The mass change is obtained by detecting the change in resonance frequency of the cantilever through the resonance driver and detection resistor, based on the resonance frequency-mass relationship of the cantilever. This enables the TGA function to be realized. The thermocouple integrated into the cantilever, based on the Seebeck effect, provides the temperature information, thus enabling the DTA function. The tiny structure of the microcantilever results in a significant reduction in the heat capacity of the chip, leading to a power responsivity at the V/W level, which is three orders of magnitude higher than that of commercial DTA instruments. Our chips achieve heating and cooling rates above 600 °C/min, which is one to two orders of magnitude higher than conventional instruments, significantly improving the efficiency of TGA and DTA measurement. Reducing the volume of sample requirements allows for uniform heating during the rapid heating process. Furthermore, the sample is loaded directly onto the thermocouple integrated into the cantilever, ensuring highly accurate temperature detection. Thus, precise and efficient on-chip MEMS TGA and DTA characterization can be realized with our cantilever. Moreover, the integrated microcantilever has dimensions on the micron scale, and the integrated on-chip heater obviates the necessity for a heating oven, thus enabling the chip to be placed under a microscope for in situ optical observation of the morphological evolutions that occur in the sample during thermal analysis. The aforementioned thermocouple-integrated resonant microcantilever chip has been employed for TGA or DTA measurements of a diverse range of materials, including metals (indium and tin standards) and calcium oxalate monohydrate (CaC_2_O_4_·H_2_O).

## Results

### Sensor design and fabrication

**Fig. 1:**
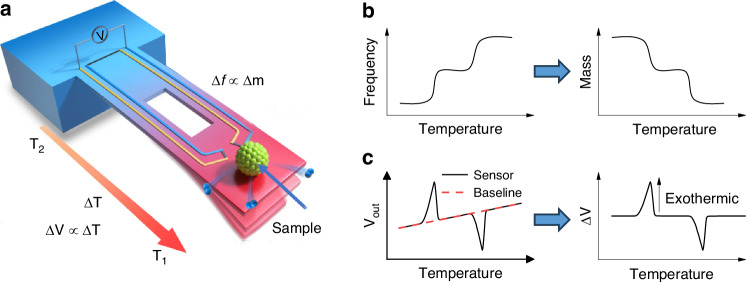
**Working principle of the integrated cantilever a** Schematic illustration of the resonant microcantilever for TGA and DTA measurement, with a core component of the cantilever for mass measuring and the thermocouples for temperature sensing. **b** Based on the Seebeck effect, the output voltage of the thermocouple is proportional to the temperature difference, thus being able to record the temperature change caused by the endothermic (or exothermic) process. **c** Based on the proportional relationship between mass change (Δ*m*) and the measured resonant frequency shift (Δ*f*) of the microcantilever, the real-time measured frequency signal can be transformed into a typical TGA curve

Figure 1a shows a schematic illustration of the integrated cantilever. The sample is placed directly onto the free end of the cantilever, which has an integrated heater for on-chip heating and cooling. Two polysilicon thermocouples are also integrated into the cantilever. The hot junction is in the sample area, while the cold junction is on the bulk silicon substrate, ensuring accurate temperature detection. To separate the high-temperature zone from the low-temperature zone, an adiabatic window is placed in the center of the cantilever to block heat transfer from the free end to the fixed end. The detection of mass changes in loaded samples is based on the resonance frequency shift of the cantilever.

As shown in Fig. 1b, we use integrated thermocouples that rely on the Seebeck effect to detect the temperature difference between hot and cold junctions^36,37^. According to the Seebeck effect, the output voltage of thermocouples can be expressed as^37,38^:

1 $$V=N\left({{\rm{\alpha }}}_{B}-{{\rm{\alpha }}}_{A}\right)\left({T}_{{hot}}-{T}_{{env}}\right)=N\left({{\rm{\alpha }}}_{B}-{{\rm{\alpha }}}_{A}\right)\left({T}_{{heat}}-{T}_{{env}}+\Delta {T}_{{loading}}\right)$$

where *N* is the number of thermocouple pairs, α_A_ and α_B_ are the Seebeck coefficient values of the two materials that form the thermocouples. *T*_hot_ is the temperature of the hot junction, which is composed of the temperature generated by the heater (*T*_heat_) and the temperature difference caused by the loaded sample (Δ*T*_loading_). T_env_ is the temperature of the environment. Therefore, we can measure the *T*_hot_ by recording the output voltage of thermocouples. To perform DTA measurement, two integrated cantilever chips with identical characteristics should be applied simultaneously under the same conditions to reduce the common mode noise and disturbances. One chip acts as a reference sensor, and the other as a test sensor for the load sample. Hence, the differential output voltage between reference and sensing can be expressed as:

2 $$\Delta V=N\left({{\rm{\alpha }}}_{B}-{{\rm{\alpha }}}_{A}\right)\left({\Delta T}_{{heat}}+\Delta {T}_{{loading}}\right)$$

The Δ*T*_heat_ is the difference between temperatures generated by heaters, caused by differences between chips in the specifications such as the heating resistance, the heat capacity, and the thermal conductance. It may be caused by some inevitable unevenness in the deposition rate and etching rate in the fabrication process. Therefore, it can be eliminated by measuring the same chips before loading the sample under the same conditions as the baseline for formal DTA measurement. This method allows us to determine the temperature difference produced by the loaded samples.

As illustrated in Fig. 1c, we utilize the frequency change of the cantilever to quantify the mass change based on the conservation of mechanical energy at the resonant cantilever. When a small mass change occurs at the free end, and the mass change is much smaller than the effective mass of the cantilever, the relationship between the mass change and the 1st mode frequency shift can be expressed as^33–35,39^.

3 $$\Delta m\approx \frac{k\Delta f}{2{\pi }^{2}{{f}_{0}}^{3}}=2{m}_{{eff}}\frac{\Delta f}{{f}_{0}}$$

where Δ*m* is the tiny mass change on the free end, *k* is the elastic coefficient of the cantilever, *f*_0_ is the resonant frequency of the cantilever before mass loading, Δ*f* is the resonant frequency shift of the cantilever, and *m*_eff_ is the effective mass. The Eq. (3) shows that a small change in mass is directly proportional to the frequency change. In our chips, we can measure the mass change in real-time by recording the frequency shift of the resonant cantilever, as shown in Fig. 1b.

**Fig. 2:**
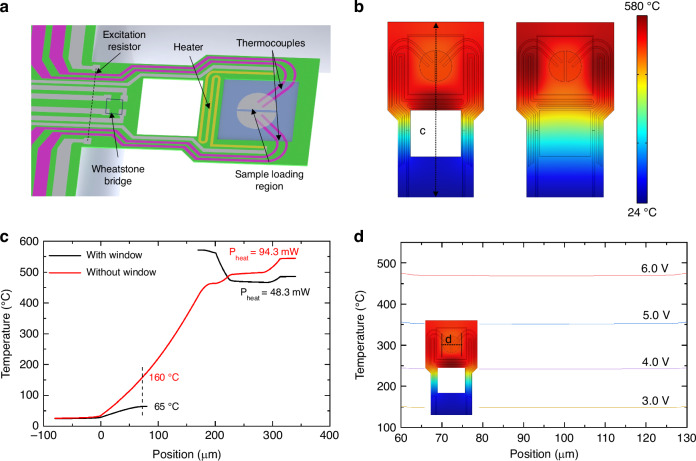
**Structure and finite element simulation results of the resonant cantilever a** 3D model of the cantilever illustrating its thermocouple, sample-loading area, and integrated heater. **b** The temperature distribution simulation results are obtained by considering key factors such as electrical heating, heat transfer, and air convection. **c** Simulation results show the hindering role of the thermal isolation window on heat conduction. **d** Simulation results of the temperature of the hot junction show good uniformity (variation within ±1%)

Figure 2a shows the detailed structure of the integrated cantilever chips. The sample-loading area is near the free end, where the surrounding bulk silicon is etched to reduce additional heat capacity. We selected molybdenum metal as the material for the heating resistor and the hot junction connection material. Molybdenum is a commonly used high-melting-point metal in MEMS fabrication. Its coefficient of thermal expansion is closely matched with that of silicon, which minimizes the risk of detachment at high temperatures. Moreover, molybdenum and silicon can form MoSi_2_ at high temperatures, which provides a stable contact resistance^40,41^. The two hot junctions of the thermocouples are covered with semicircular molybdenum to ensure a uniform temperature across the sample region. For high-temperature sensitivity, we use n+ and p+ polysilicon to fabricate the thermocouple, which has a Seebeck coefficient significantly higher than the commonly used metals in IC fabrication^15,36^. In order to achieve both integrated resonance excitation and frequency readout functions, a silicon resistor is designed near the fixed end of the cantilever for electrothermal driving. Additionally, a Wheatstone bridge composed of silicon piezoresistors is fabricated near the fixed end to facilitate resonant frequency readout^33,39^. Due to the limitations of silicon resistors, which may not function properly at temperatures exceeding 125 °C^35,42^, a thermal isolation window has been designed to separate the heater and piezoresistors. This design blocks direct thermal conduction within the cantilever while improving thermal resistance from the sample area to the surrounding environment. As a result, the design of the thermal isolation window helps to increase the power responsivity of the system.

We then perform finite element simulation to model the temperature distribution of the integrated cantilever using COMSOL Multiphysics analysis software. The software modeled the thermoelectrically generated heat using the electric current interface. The element also accounts for convective heat transfer in the air atmosphere, and a fixed convective heat dissipation coefficient is set to reduce simulation time without compromising accuracy. The amplitude of cantilever beam resonance is typically at the nanometer scale, resulting in a negligible effect on the convective heat transfer coefficient. Consequently, this simulation does not account for temperature variations caused by vibrations. The thermal conductivity of single-crystal silicon is adjusted for temperature based on the literature^43^ to improve accuracy at high temperatures. Figure 2b illustrates the simulation results for the overall temperature distribution on the surface of the cantilever when the temperature at the center of the sample region reaches 500 °C. Additionally, we conducted a simulation to analyze the impact of the thermal isolation window, which blocks heat transfer from the free end to the fixed end. Figure 2c presents the effect of the thermal isolation window on the temperature distribution along the center axis of the cantilever. With the isolation window, the temperature around the Wheatstone bridge remains below 65 °C as the temperature of the free end exceeds 500 °C, at a heating power of 48.3 mW. In contrast, without the isolation window, under similar free-end temperature conditions (at a heating power of 94.3 mW), the temperature around the Wheatstone bridge increases to 160 °C, which could lead to p-n junction leakage. The simulation results demonstrate effective thermal isolation between the free and fixed ends, protecting the resonant excitation and readout silicon resistors at high operating temperatures. In Fig. 2d, the temperature distribution of the sample-loading region along the horizontal axis is shown at different heating voltages. The simulation results indicate a uniform temperature distribution in the center of the sample-loading region, with a variation of within ±1%.

**Fig. 3:**
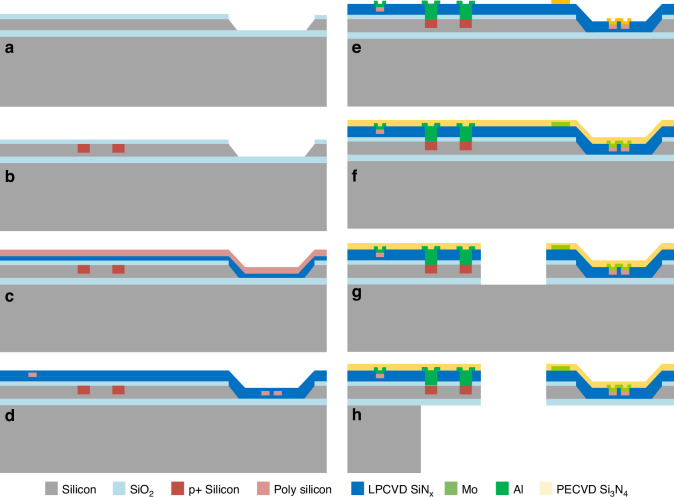
**Fabrication process of the integrated cantilever chips a** KOH etching of sample-loading region after thermal oxidation. **b** Ion-implantation of resonant excitation and readout resistance. **c** LPCVD SiN_x_ and polysilicon deposition. **d** Fabrication of thermocouples through ion-implantation, diffusion, and RIE, and then depositing LPCVD SiN_x_. **e** Patterning of Mo heater and Al electric connection. **f** PECVD film deposition for antioxidant protection. **g** DRIE for the device layer to pattern the shape of the cantilever. **h** DRIE is used to handle the layer and release the cantilever

Our chips are produced on 4-inch (100) SOI wafers, enabling precise control over the cantilever layer thickness of 3 μm, a handle layer thickness of 500 μm, and a box layer thickness of 700 nm. The detailed fabrication process is shown in Fig. 3: (a) The thermal SiO_2_ of 350 nm is fabricated through a heating oxidation furnace. After photolithography and RIE etch of SiO_2_, a pool is defined by KOH wet etching for the sample-loading region. (b) The silicon resistors of resonant excitation and readout resistors (Wheatstone bridge) are defined by photolithography, ion-implantation, and diffusion. (c, d) After depositing 200 nm of SiN_x_ and 500 nm of polysilicon films by LPCVD, the main part of polysilicon thermocouples is fabricated through ion implantation, diffusion, and RIE sequentially. A 300 nm SiN_x_ film is then deposited to protect the thermocouples. (e) After etching the connection window of the polysilicon thermocouple and silicon resistors, the Mo films are patterned using the lift-off process to create microheaters and connect the thermocouples. Subsequently, the Al films are patterned using the lift-off process to establish electrical connections for the silicon resistors. (f) Depositing a 200 nm PECVD Si_3_N_4_ layer provides protection against oxidation and electrical isolation. (g) A RIE process of dielectric layers is followed by DRIE of the device layer and RIE of the box layer to form the shape of the resonant cantilever. (h) The handle layer is removed from the back side using a DRIE process, thus releasing the cantilever.

### Characterization of the differential thermopiles

**Fig. 4:**
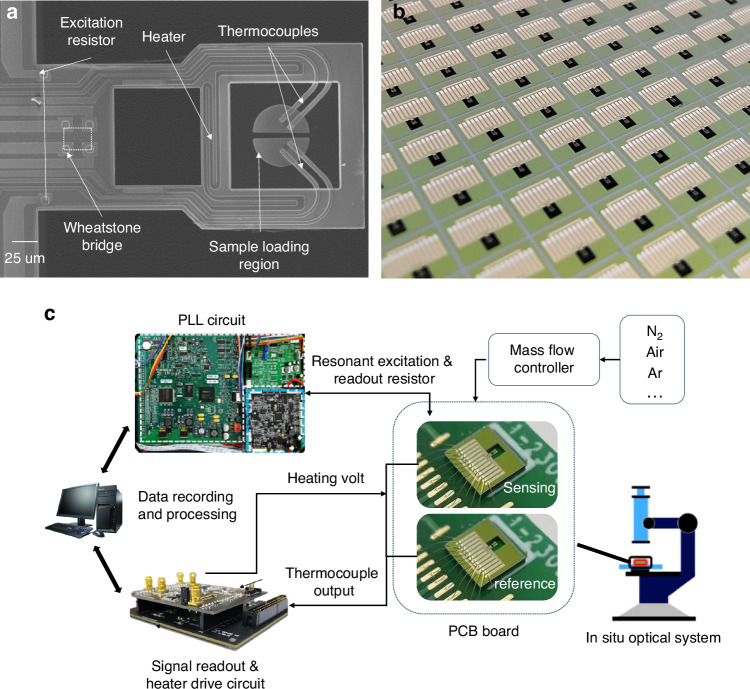
**Image of the integrated cantilever and schematic diagram of the testing system a** SEM image of the cantilever, showing the detail of the integrated component. **b** Microcantilevers fabricated on 4-inch SOI wafers. **c** Schematic diagram of the test system for TGA and DTA measurements

Figure 4a depicts the morphological image of the manufactured resonant cantilevers. The cantilever measures 340 μm in length, 190 μm in width, and 3 μm in thickness. The sample-loading area has a diameter of 60 μm, and the thermal isolation window measures 100 μm × 90 μm. The SEM image of the cantilever in Fig. 4a clearly reveals the structure and morphology of the integrated thermocouple, heater, resonant excitation/readout resistor, and sample-loading area. Figure 4b illustrates the wafer-level fabrication of the cantilever chips on a 4-inch SOI wafer. The dimensions of a single chip are 2.7 mm by 2.3 mm. A single 4-inch SOI silicon wafer can be used to mass-produce 990 chips, with a yield rate exceeding 70% Fig. 4c schematically depicts the test system for TGA and DTA measurements. The resonant excitation and readout resistors of the sensing chip are connected to a phase-locked-loop (PLL) circuit for tracking the resonant frequency. The thermocouples of the two chips are connected to the signal readout circuit to measure the output voltage. The heating drive circuit uses PID control to adjust the drive voltage of the heating resistor based on the user-set temperature and the thermocouple output, thereby enabling programmed temperature control. Both circuits are linked to a computer for data recording and further processing. We use a sample-loading system consisting of a microscope, micropump, and microneedle to load samples onto the device. First, the sample to be loaded is prepared as a solution or suspension. The liquid is then drawn up through the microneedle connected to the micropump and then dispensed onto the surface of the cantilever under the microscope. Finally, the solution evaporates, leaving the sample for analysis. It is worth noting that, similar to crucibles, each chip is typically used to measure only one type of sample to avoid cross-contamination. If necessary, ultrasonic cleaning can be employed to remove the sample from the surface of the cantilever.

**Fig. 5:**
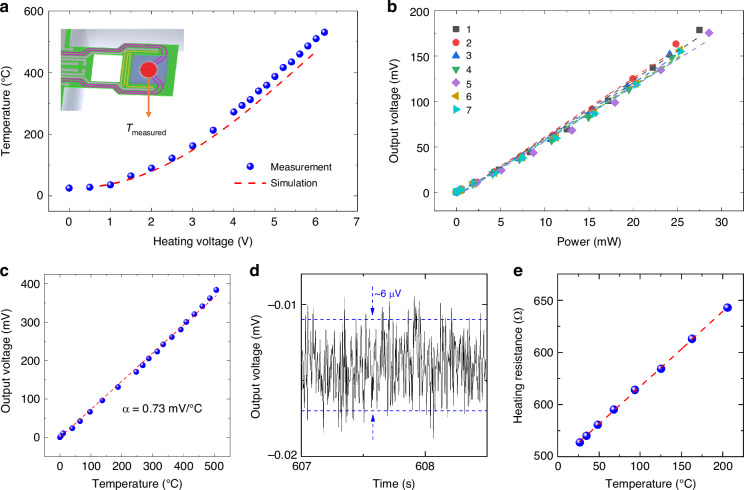
**Test results for the thermal properties of microcantilevers a** The temperature at the hot junction is measured under different heating voltages, and the result is in agreement with the simulation. **b** The output voltage of the thermocouple at various heating powers indicates a high power response of 6.1 V/W. **c** The hot junction output voltage vs. temperature shows a temperature response of 0.73 mV/°C. **d** The output voltage noise of the integrated thermocouples generated by the test circuit. **e** The relationship between the heating voltage and the heating resistance indicates that the TCR is 0.0014/°C

The temperature dependence of the chip was first assessed by measuring the temperatures of the hot junction at various heating voltages using a non-contact infrared thermal imager with a spatial resolution of 20 μm. Simultaneously, the test system recorded the output voltage of the thermocouples. Figure 5a shows the measured and simulated temperatures of the sample-loading area as a function of the heating voltage, and the agreement is very good, indicating that our simulation is reliable. The sample region can be heated up to 530 °C under a heating voltage of 6.2 V. Meanwhile, Fig. 5b shows the corresponding relationship between the output voltage and heating power of the seven chips. The average power response of each chip is 6.1 V/W, which is three orders of magnitude higher than that of traditional instruments (generally in the mV/W level)^18,19^, and the relative standard deviation is only 3%, indicating strong consistency between different chips. In Fig. 5c, the output voltage of thermocouples is shown for different measured temperatures. The output voltage demonstrates a linear response to the temperature, with a high-temperature responsivity of 0.73 mV/K. The root-mean-square (*rms*) voltage noise of the MEMS thermocouples can be expressed as follows:

4 $${V}_{{noise},{rms}}=\sqrt{4{k}_{B}{TRB}}$$

where *k*_B_ is the Boltzmann’s constant, *T* is the room temperature, *R* is the thermocouples resistance (10kΩ), and *B* is the system bandwidth (400 Hz)^22,23^. According to Eq. (3), we can have a *rms* voltage noise of 0.26 μV for our chips. Therefore, we can calculate a noise equivalent temperature (NET) of 2.8 mK, based on the 8 × *V*_noise,rms_, and temperature responsivity^23^. As shown in Fig. 5d, the voltage noise generated by our test circuit is 6 μV, corresponding to an equivalent temperature resolution of 66 mK. We also measure the change in heating resistance with the working temperature, as illustrated in Fig. 5e, indicating a TCR (temperature coefficient of resistance) of 0.0014/K, due to temperature variations over the cantilever. Moreover, we compared the thermocouple output voltage of the cantilever in the stationary state with that of the same cantilever in a resonant state under different temperatures. The result is shown in Fig. S1. It was observed that the variation in output voltage was considerably less than the output voltage noise. Therefore, temperature changes due to resonance were not considered in the practical measurement.

**Fig. 6:**
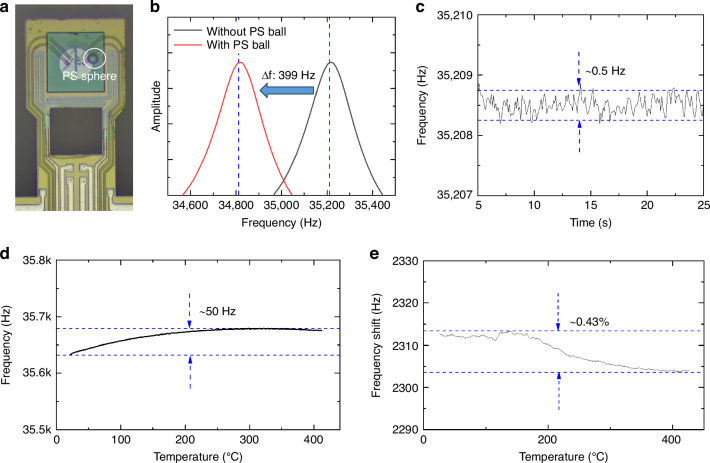
**Mass sensitivity of the microcantilever a** Optical images of the cantilever before and after loading with a standard PS sphere. **b** Based on the frequency and amplitude of the cantilever before and after loading with a standard PS sphere, the mass sensitivity of the cantilever can be tested to be 0.09 Hz/pg. **c** The noise floor is approximately 0.5 Hz, resulting in a mass resolution of 5.5 pg. **d** Frequency variation with operating temperature. Within the tested temperature range, the maximum frequency shift is 50 Hz. **e** The frequency difference generated by the loaded silicon powder varies with temperature. Within the tested temperature range, the maximum frequency shift is 0.43%

Then, the mass-sensing properties of the fabricated cantilevers are calibrated. A flowing DC-biased AC is applied to the excitation resistor, and the Wheatstone bridge is also connected to the PLL interface circuit. In this way, we can measure the real-time frequency shift. The resonant frequency of our cantilever is approximately 35 kHz, with a *Q* factor of 190. To calibrate the mass sensitivity of our cantilever, we measure the resonant frequency of the cantilever before and after placing a standard polystyrene (PS) sphere on the sample region, as illustrated in Fig. 6a, b. The diameter of the standard PS sphere is 20 μm, and the density is 1.05 g/cm^3^. A resonant frequency change of 399 Hz is measured after loading the standard PS sphere, which leads to a mass responsivity of 0.090 Hz/pg for the cantilever. The noise floor of the frequency signal is approximately 0.5 Hz, leading to a mass resolution of 5.5 pg. Therefore, our chips can conduct thermogravimetric analysis with ng-level samples. When the sample region of the cantilever is heated from room temperature to 400 °C, only a slight frequency shift of 50 Hz is measured, equivalent to a mass change of 0.56 ng. This shift is caused by a combination of factors; on the one hand, the Young’s modulus of the materials that make up the cantilever beam varies with temperature, and on the other hand, thermal expansion leads to changes in the cantilever beam structure^44^. We measured the variation of mass sensitivity with temperature. As shown in Fig. 6e, by comparing the frequencies of an empty cantilever with those of the same cantilever loaded with silicon powder at different temperatures, we found that the frequency difference varied by only 0.43% as the temperature varied from room temperature to 400 °C. Therefore, we consider the variation in mass sensitivity with temperature to be negligible.

### DTA measurement using integrated cantilever chips

Metal standards with a fixed melting point are commonly used to calibrate DTA measurements. In this work, we have selected indium (In) and tin (Sn) standards to validate our DTA measurement performance. As mentioned above, two chips are used simultaneously in DTA measurement. One chip contains a sample, while the other is left empty. The DTA measurements are carried out at various heating rates in an air atmosphere. Because our cantilevers are small, we place them under a microscope to observe the morphological changes of the samples in situ during the DTA measurements.

**Fig. 7:**
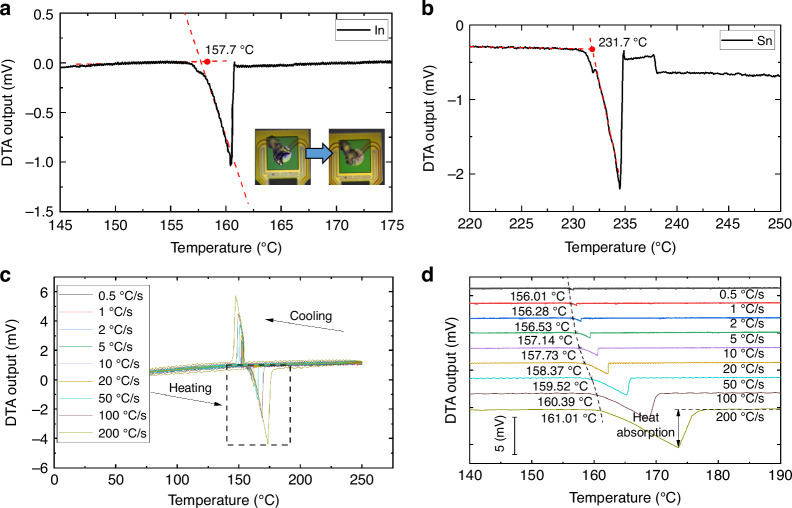
**Differential thermal analysis results of metal standards** DTA curves of the In (**a**) and Sn (**b**) standards melting under a heating rate of 10 °C/s. The measured melting points of In and Sn are consistent with the reported values. The in situ optical images in insets in a depict the process of indium melting. **c** DTA curves of indium melting and resolidification at heating/cooling rates from 0.5 °C/s to 200 °C/s. **d** The melting points of indium are measured using DTA curves at various heating rates

**Fig. 8:**
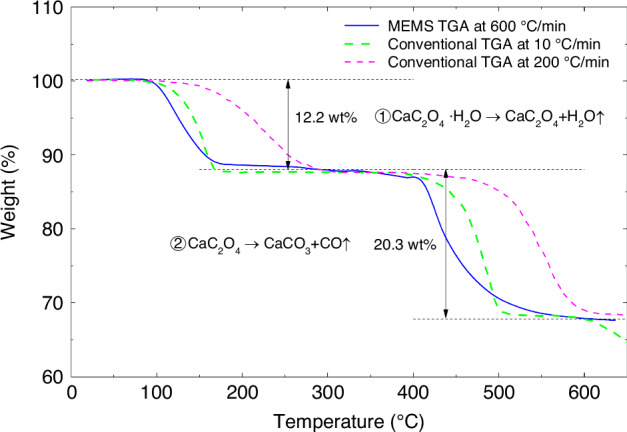
**Comparison of thermogravimetric analysis results of CaC**_**2**_**O**_**4**_**·H**_**2**_**O using the cantilever in this work and the conventional instrument** The TGA curves show that the decomposition process of CaC_2_O_4_·H_2_O sample can be divided into two stages. At high heating rates, the thermal hysteresis of our cantilever is negligible compared with the commercial instrument

We first measure the melting process of In and Sn at a heating rate of 10 °C/s. The results are shown in Fig. 7a, b. We can observe the sharp heat absorption peak caused by the melting process. The video in Supporting Material S1 and the insets in Fig. 7a show the in situ optical images of the indium melting process. The indium undergoes a noticeable morphological change after reaching its melting point. Based on the heat absorption peak in the measured DTA curve, we can calculate the melting point of the metal using our chips^45^. The measured melting point of indium is 157.7 °C, which is only 1.1 °C higher than the standard value of 156.5 °C in literature^45,46^. As for tin, the melting point is measured as 231.7 °C, only 0.2 °C smaller than the standard valve (231.9 °C)^46^. The results presented in Fig. 8a, b confirm the temperature accuracy of our chips.

We further test the indium melting and solidification process at different heating and cooling rates from 1 °C /s to 200 °C/s. The heat absorption and release during melting and solidification processes induce sharp peaks in the DTA curves, as shown in Fig. 7c. As the rate of temperature rise and fall increases, the DTA signal becomes larger, thereby facilitating the measurement of small heat absorption or exothermic processes. We also calculate the melting point of indium at different heating rates, as shown in Fig. 7d. The melting point at 200 °C/s exhibits only a 5 °C difference compared to the melting point at 1 °C/s. The results demonstrate that the chip exhibits small thermal hysteresis even at rapid temperature changes.

### TGA measurement using integrated cantilever chips

To demonstrate the technical merit of our integrated cantilever in TGA measurement, inorganic crystals with known molecular structures are selected. CaC_2_O_4_ ∙ H_2_O is a standard substance for evaluating TGA measurement. The TGA measurement by our cantilevers is performed at a heating rate of 600 °C/min in an air atmosphere. We also measure the same sample with a conventional TGA instrument at heating rates of 10 °C/min and 200 °C/min. The results are plotted together in Fig. 8 for comparison. Two stages of decomposition can be observed in the TGA curves measured from room temperature to 620 °C. The first stage can be assigned to the loss of water of crystallization with a weight loss of 12.2%. The second stage shows a weight loss of 20.3%, which indicates CaC_2_O_4_ decomposed into CaCO_3_. Both decomposition-induced weight losses are consistent with the theoretical values (12.32%, 19.16%)^42,47^. When the CaC_2_O_4_ ∙ H_2_O sample was analyzed using a conventional TGA instrument (NETZSCH TG 209 F1 Libra), the TGA curve obtained at a heating rate of 200 °C/min showed a significant hysteresis compared to the test at a heating rate of 10 °C/min. Fortunately, even when heating at a high rate of 600 °C/min, the TGA results obtained with the cantilever closely match those obtained with a conventional TGA instrument using a heating rate of 10 °C/min. The results suggest that the thermal hysteresis phenomenon of the cantilever is minimal in MEMS TGA analysis, and it dramatically improves the efficiency of the TGA analysis.

## Conclusion

In summary, we have developed and fabricated an integrated resonant microcantilever for performing both thermogravimetric analysis (TGA) and differential thermal analysis (DTA) measurements. Each microcantilever chip includes a microheater, resonance excitation/readout resistors, and thermocouples to enable controlled heating, precise mass detection at the picogram level, and accurate temperature measurement. Our cantilevers show a power responsivity of 6.1 V/W, temperature sensitivity of 0.73 mV/ °C, and temperature resolution of 2.8 mK. Our cantilevers also have a mass sensitivity of 0.09 Hz/pg and a mass resolution of 5.5 pg. We then performed TGA measurements on CaC_2_O_4_ ∙ H_2_O and DTA measurements on indium and tin metal standards. Our test results demonstrate that our chip can perform high-precision TGA and DTA analysis. Our chip has faster heating/cooling speeds and consumes fewer samples than conventional TGA and DTA instruments. Our integrated cantilever device is expected to enable efficient TGA and DTA testing in various essential application areas in physics, chemistry, metallurgy, pharmaceuticals, and nanotechnology.

## Supplementary information

Supplemental Material

Video of the indium melting during DTA measurement using our chips
