# Supplementary material for: Thermocouple-integrated resonant microcantilever for on-chip thermogravimetric (TG) and differential thermal analysis (DTA) dual characterization applications
Source: Microsyst Nanoeng. 2025 Mar 26;11:54. doi: 10.1038/s41378-024-00828-9 (PMC11937515; doi:10.1038/s41378-024-00828-9)
Supplement: Supplementary file 1 — Supplemental Material [file 41378_2024_828_MOESM1_ESM.docx]

Thermocouple-Integrated Resonant Microcantilever for On-chip Thermogravimetric (TG) and Differential Thermal Analysis (DTA) Dual Characterization Applications

*Yuhang Yang^1,2^, Hao Jia^1,2^, Zechun Li^1,2^, Zhi Cao^3^, Haozhi Zhang^1,2^, Pengcheng Xu^1,2^, and Xinxin Li^1,2^*

^1^ State Key Lab of Transducer Technology, Shanghai Institute of Microsystem and Information Technology, Chinese Academy of Sciences, Shanghai 200050, CHINA,

^2^ University of Chinese Academy of Sciences, Beijing 100049, CHINA,

^3^ School of Chemical and Environmental Engineering, Shanghai Institute of Technology, Shanghai 201418, CHINA

*Email: [xpc@mail.sim.ac.cn](mailto:xpc@mail.sim.ac.cn); [xxli@mail.sim.ac.cn](mailto:xxli@mail.sim.ac.cn)

We heated the cantilever to different temperatures at stationary state, then scanning the resonance frequency, and finally kept the cantilever in resonance by the PLL circuit. At the same time the thermocouple output changes are recorded throughout the process. As shown in Fig. S1, the output voltage shifts are less than the resolution of the testing system. So, we do not consider temperature variations caused by resonance in our actual measurements
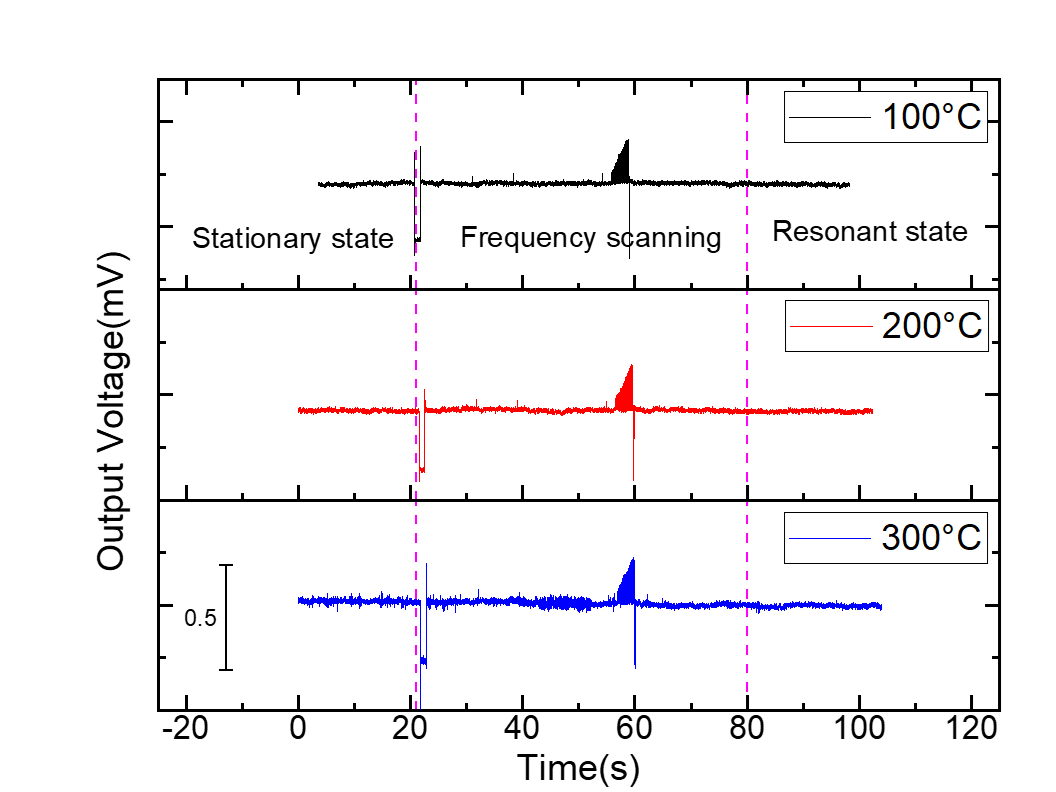


**Fig. S1 Variation of thermocouple output of the cantilever from stationary state to resonant state at different temperatures.**
